# Direct Oral Anticoagulants vs. Vitamin K Antagonists for Atrial Fibrillation in Cardiac Amyloidosis: A Systematic Review and Meta-analysis

**DOI:** 10.31083/RCM26948

**Published:** 2025-03-13

**Authors:** Qinling Nong, Shucheng Liang, Wengen Zhu, Yili Chen, Tang Zhang

**Affiliations:** ^1^Department of Cardiology, Ruikang Hospital Affiliated to Guangxi University of Traditional Chinese Medicine, 530000 Nanning, Guangxi, China; ^2^Faculty of Medicine, Macau University of Science and Technology, 999078 Macau SAR, China; ^3^Department of Cardiology, The First Affiliated Hospital of Sun Yat-Sen University, 510060 Guangzhou, Guangdong, China; ^4^Department of Cardiology, The Second Affiliated Hospital of Guangxi Medical University, 530003 Nanning, Guangxi, China

**Keywords:** direct oral anticoagulants, vitamin K antagonists, atrial fibrillation, cardiac amyloidosis, outcome

## Abstract

**Background:**

This study aimed to systematically review and synthesize evidence comparing direct oral anticoagulants (DOACs) with vitamin K antagonists (VKAs) for anticoagulation in patients with atrial fibrillation (AF) and cardiac amyloidosis (CA).

**Methods:**

A comprehensive search of PubMed and EMBASE databases was conducted through January 2024 to identify studies comparing DOACs and VKAs in AF patients with CA. Eligible studies underwent rigorous screening and data extraction to evaluate safety and efficacy outcomes.

**Results:**

Four studies met the criteria. The first study reported similar embolic event rates between DOACs (3.9%) and VKAs (2.9%) per 100 patient years, while major bleeding rates were 5.21% and 3.74%, respectively. The second paper found stroke rates of 2% for DOACs and 4% for VKAs, with bleeding complications observed in 10% of DOAC patients compared to 20% in VKA patients. The third cohort demonstrated that DOACs were associated with significantly lower risks of stroke and major bleeding compared to VKAs. The last study reported embolic event rates of 1.6 and 2.0 per 100 patient years for DOACs and VKAs, respectively. In the pooled analysis, DOACs were associated with a reduced risk of thromboembolic events (odds ratio [OR] = 0.52; 95% confidence interval [CI]: 0.32–0.84), and no difference in major bleeding between the two groups (OR = 0.61, 95% CI: 0.25–1.51).

**Conclusions:**

Existing studies support the use of DOACs as a non-inferior therapeutic option compared to VKAs for preventing thromboembolism in patients with AF and cardiac amyloidosis. DOACs may also offer practical advantages, including reduced bleeding risks and ease of management, but further high-quality randomized controlled trials are needed to confirm these findings and guide clinical practice.

## 1. Introduction

Amyloidosis is a rare group of disorders characterized by the pathological deposition of amyloid fibrils, resulting in from misfolded proteins, within the intracellular and/or extracellular compartments of a variety of tissues and organs including the heart, kidneys, central nervous system, and digestive system [1]. Recent advances in non-invasive diagnostic modalities, such as cardiac magnetic resonance imaging and bone scintigraphy, have revolutionized the detection of cardiac amyloidosis, particularly transthyretin amyloid cardiomyopathy (ATTR-CM) [2]. These technologies have significantly increased diagnostic accuracy and recognition rates of this previously underdiagnosed condition [3]. Cardiac amyloidosis (CA) encompasses several types, with ATTR-CM and amyloid light-chain (AL) being the most common. ATTR-CM can be further classified into two subtypes, namely wild-type (ATTRwt) and hereditary ATTR (hATTR) amyloidosis [4].

CA primarily manifests as restrictive cardiomyopathy [5], characterized by signs and symptoms of right ventricular diastolic dysfunction, such as elevated jugular venous pressure, ascites, peripheral edema, and dyspnea. In CA patients, amyloid infiltration of the atrial myocardium leads to complex structural and electrical remodeling of the atria, increasing the risk of atrial fibrillation (AF) and predisposing patients to thromboembolism due to altered electrophysiological characteristics [6]. A previous study has reported a high prevalence of AF in CA patients, with rates of up to 40% in wtATTR and 11% in hATTR [7]. Therefore, preventing the formation of left atrial thrombus is crucial in CA patients with AF to reduce the risk of cardioembolic stroke.

Direct oral anticoagulants (DOACs), such as rivaroxaban, apixaban, dabigatran, and edoxaban, are newer anticoagulants that have gained recognition as viable alternatives to vitamin K antagonists (VKAs) such as warfarin. Recent guidelines from the American Heart Association [8] and the European Society of Cardiology [9] have shifted the preference towards DOACs. Currently, anticoagulation therapy is recommended for AF patients with CA, regardless of their CHA₂DS₂-VASc (Congestive heart failure, Hypertension, Age ≥75 years, Diabetes mellitus, Stroke or transient ischemic attack history, Vascular disease, Age 65–74 years, and Sex category) score [10]. However, managing anticoagulation in this population is challenging due to high bleeding risks and frequent renal impairment, complicating clinical decision-making.

There is a lack of comprehensive studies that directly compare the effectiveness and safety of DOACs with VKAs in AF patients with CA. To address this gap, our current systematic review aimed to comprehensively analyze the existing research on the use of DOACs compared with VKAs in patients with AF and CA.

## 2. Methods

Our systematic review followed the guidelines outlined in the Preferred Reporting Items for Systematic Reviews and Meta-Analyses (PRISMA).

### 2.1 Eligibility Criteria

The following eligibility criteria were applied. Studies were included if they: (1) focused on patients with both AF and CA, (2) compared DOACs with VKAs for safety and efficacy, and (3) were published in English. Studies were excluded if they: (1) involved other forms of anticoagulation therapy not relevant to DOACs or VKAs, (2) were case reports or reviews rather than observational studies or randomized controlled trials, or (3) did not report on relevant outcomes such as thromboembolic or bleeding events.

### 2.2 Literature Search

A systematic independent literature search was performed by 2 reviewers in the EMBASE and PubMed databases until January 2024 to identify relevant articles that examined and analyzed the effectiveness and safety of DOACs as compared to VKAs in patients with AF and CA. The search employed a combination of the following keywords: (1) “amyloidosis” OR “ATTR-CM” OR “transthyretin amyloid cardiomyopathy” OR “ATTR CA” or “light chain amyloid cardiomyopathy”, and (2) “anticoagulants” OR “anticoagulation”. We applied the English language restriction in the literature search.

### 2.3 Study Selection

The initial search was conducted on PubMed and EMBASE databases. Retrieved studies were screened based on their titles and abstracts, and relevant studies were subjected to full-text reading. Studies that fulfilled the eligibility criteria mentioned above were included. Disagreements between the authors were resolved by discussion, or by seeking input from a more senior expert.

### 2.4 Data Extraction

The data extracted from the included studies consisted of the author’s name, year of publication, and baseline characteristics such as age, sex renal function, CHA_2_DS_2_-VASc score, HAS-BLED score (Hypertension, Abnormal renal/liver function, Stroke, Bleeding history, Labile international normalized ratio, Elderly age, Drugs/alcohol use), prior disease, study treatment, CA subtypes, study design, effectiveness, and safety outcome results.

### 2.5 Statistical Analysis

The quality of each observational study was assessed using the Newcastle-Ottawa Scale (NOS) tool [11]. To evaluate heterogeneity, we conducted a Cochrane Q test and calculated the I^2^ statistic, with results expressed as *p*-values and I^2^ values. Initially, we conducted a narrative review. For the quantitative analysis, we used a Mantel-Haenszel random-effects model to account for potential heterogeneity across the included studies, with analyses performed using Review Manager software (version 5.4, The Cochrane Collaboration, Nordic Cochrane Center, Copenhagen, Denmark). Effect measures were reported as odds ratios (ORs) with 95% confidence intervals (CIs). Subgroup and sensitivity analyses were not conducted due to the limited number of studies included. In line with the Cochrane Handbook guidelines [12], publication bias was not assessed using a funnel plot, as fewer than 10 studies were included in this review.

### 2.6 Protocol Registration

This meta-analysis was not registered in a public database. The authors acknowledge the importance of protocol registration for transparency and reproducibility.

## 3. Results

### 3.1 Study Selection

**Fig. 1.**
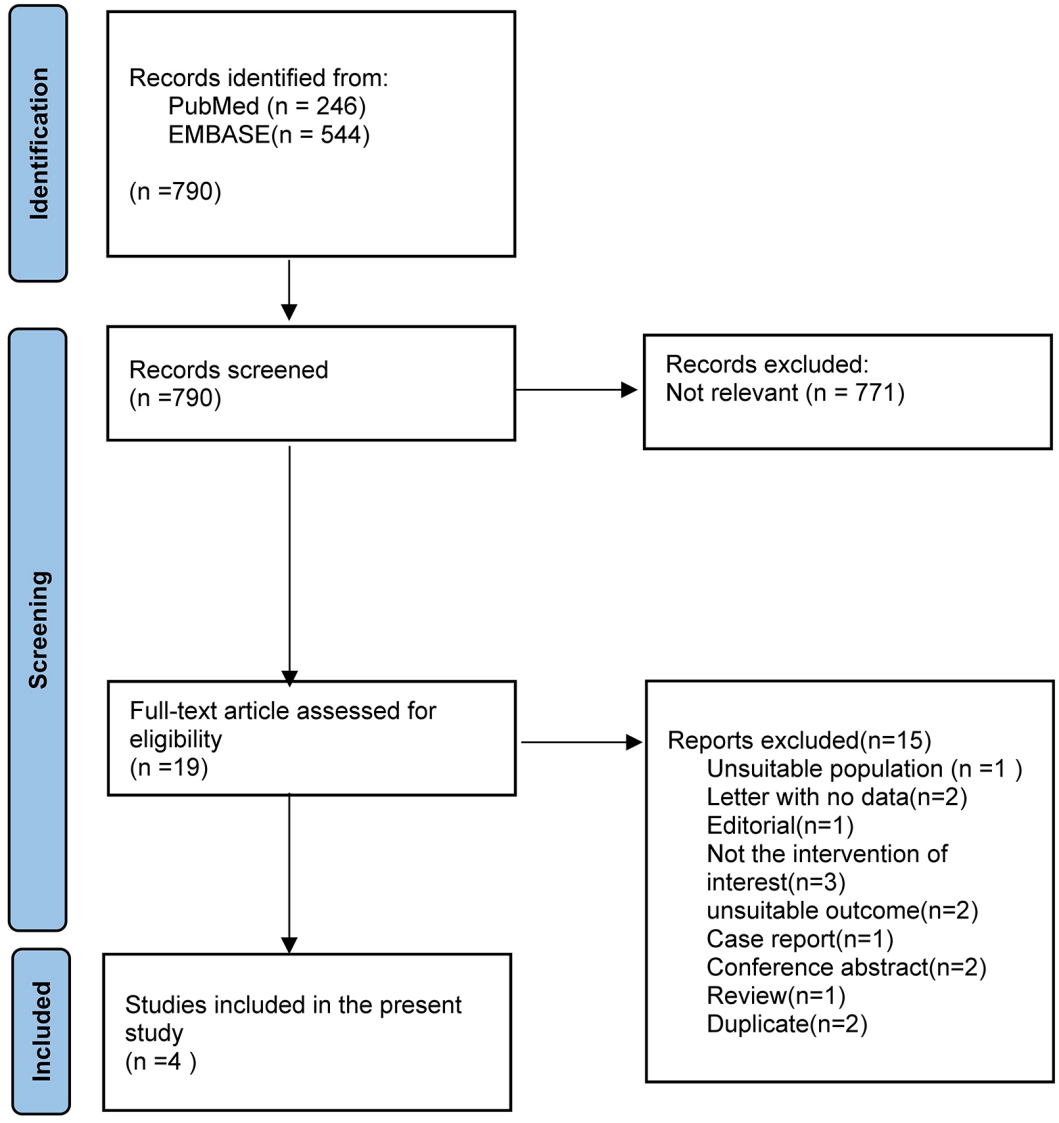
**Overview of the meta-analysis search and selection process of this review**. A detailed flow diagram illustrating the study selection process for this meta-analysis, including initial database search results from PubMed and EMBASE, title and abstract screening, full-text review, and the final inclusion of studies meeting eligibility criteria.

The search and selection process is visually summarized in Fig. 1. Initially, a total of 790 articles were identified for screening, with 246 articles from the PubMed database and 544 articles from the EMBASE database. Following a title and abstract screening, 19 studies were selected for full-text review. Of these, 15 articles were excluded, leaving a total of 4 studies [13, 14, 15, 16] which met the criteria for our analysis. Detailed information on the study design and baseline characteristics of the included studies is provided in Table 1 (Ref. [13, 14, 15, 16]).

**Table 1.** **Baseline characteristics of studies included in this review**.

Author/Year	Study treatment	Study population	Amyloidosis subtype	Study design	Baseline characteristics of the population	Efficacy outcome results	Safety outcome results	Newcastle-Ottawa Scale
Mentias *et al*. [16], 2022	Apixaban (n = 238), Rivaroxaban (n = 70), Dabigatran (n = 30) vs. warfarin (n = 213)	AF patients	NA	Single center, retrospective cohort study	Age: DOACs (77.6 years) vs. Warfarin (77.0 years);	Stroke: DOACs vs. warfarin (adjusted HR 0.44, 95% CI 0.28 to 0.70)	Major bleeding: DOACs vs. warfarin (adjusted HR 0.55, 95% CI 0.36 to 0.84)	6 points
					CHA₂DS₂-VASc score: DOACs (5) vs. warfarin (5);			
					Kidney disease: DOACs (36.4%) vs. warfarin (39.4%);			
					Previous stroke: DOACs (9.1%) vs. warfarin (11.7%)			
Mitrani *et al*. [14], 2021	Apixaban (n = 60), Rivaroxaban (n = 45), dabigatran (n = 10) and other (n = 1) vs. warfarin (n = 78)	AF and flutter patients	hATTR (n = 38) and ATTRwt (n = 156)	Multicenter retrospective cohort study	Age: DOACs (75.3 years) vs. warfarin (75.2 years), CHA_2_DS_2_-VASc score: DOACs (3.8) vs. warfarin (3.7), HAS-BLED: DOACs (2.5) vs. warfarin (3.1), CKD stage IV-V: DOACs (6) vs. warfarin (7)	Embolic event rate: DOACs (3.9/100 person years) vs. warfarin (2.9/100 person years)	Major bleeding event rate: DOACs (5.21/100 person years) vs. warfarin (3.74/100 person years)	7 points
						Stroke: DOACs (3) vs. warfarin (5)		
Cariou *et al*. [15], 2021	Apixaban (n = 77), rivaroxaban (n = 35), dabigatran (n = 14) vs. warfarin (n = 81), fluindione (n = 67), Acenocoumarol (n = 2) and others (n = 3)	Atrial arrythmia patients (98% AF)	AL (25%), ATTRwt (66%), hATTR (9%)	Single center, retrospective cohort study	Age: DOACs (79 years) vs. VKA (77 years); CHA₂DS₂-VASc score: DOACs (4) vs. VKA (4); GFR: DOACs (114 mL/min) vs. VKA (183 mL/min)	Stroke events: DOACs (2%) vs. VKA (4%)	Bleeding event: DOACs (10%) vs. VKA (20%)	7 points
Vilches *et al*. [13], 2022	DOACs (n = 239) vs. VKA (n = 322)	AF	ATTRwt (83.1%), hATTR (16.9%)	Multicenter, longitudinal cohort study	Age: DOACs (77.3 years) vs. VKA (77.8 years); prior embolism: DOACs (n = 38) vs. VKA (n = 53); HAS-BLED: DOACs (2) vs. VKA (2)	Embolic events incidence rate: DOACs (1.6/100 patient years) vs. VKA (2/100 patient years)	Bleeding event: DOACs (5.1/100 person years) vs. warfarin (3.2/100 person years)	8 points

AF, atrial fibrillation; AL, amyloid light-chain; hATTR, hereditary transthyretin amyloidosis; ATTRwt, wild type transthyretin amyloidosis; DOACs, direct oral anticoagulants; VKA, vitamin K antagonist; HR, hazard ratio; CKD, chronic kidney disease; GFR, glomerular filtration rate; HAS-BLED, Hypertension, Abnormal renal/liver function, Stroke, Bleeding history, Labile international normalized ratio, Elderly age, Drugs/alcohol use; CHA_2_DS_2_-VASc, Congestive heart failure, Hypertension, Age ≥75 years, Diabetes mellitus, Stroke or transient ischemic attack history, Vascular disease, Age 65–74 years, and Sex category; NA, not available.

All four studies were retrospective cohort studies. Two of these were single-center studies, while the other two were multi-center studies. Two studies (Mitrani *et al*. [14] and Vilches *et al*. [13]) included populations with only amyloid transthyretin (ATTR), whereas the study by Cariou *et al*. [15] included a mixed population of AL, hATTR, and ATTRwt. The study by Mentias *et al*. [16] did not stratify participants by amyloidosis subtype.

### 3.2 Narrative Analysis: DOACs versus VKAs in Patients with CA

In the study by Mitrani *et al*. [14], a cohort of 290 patients diagnosed with ATTR CA between December 2001 and February 2019 were evaluated. Of these, 217 patients presented with either AF at baseline or were subsequently diagnosed with AF. Treatment included DOACs in 116 patients and warfarin in 78. Embolic events occurred at a rate of 3.9 per 100 patient-years in the DOAC group compared to 2.9 per 100 patient-years in the warfarin group (*p* = 0.74). Major bleeding events were observed in 21 patients, with event rates of 5.21 per 100 patient-years in the DOAC group and 3.74 per 100 patient-years in the warfarin group (*p* = 0.45).

In a French single-center trial carried out by Cariou *et al*. [15], 273 CA patients with atrial arrhythmia were enrolled between January 2012 and July 2020. Patients received anticoagulation with either VKAs (n = 147) or DOACs (n = 126). The VKA group exhibited poorer renal and cardiac function and included a higher proportion of patients in New York Heart Association (NYHA) class IV. Patients treated with DOAC were predominantly diagnosed with ATTRwt, whereas AL patients were more likely to be treated with VKAs. No significant difference was found in stroke rates between DOACs (2%) and VKAs (4%). However, bleeding complications were more common in the VKA group (20%) than in the DOAC group (10%). The VKA group also showed a higher rate of all-cause mortality, but after adjusting for age, NYHA class, N-terminal pro-B-type natriuretic peptide (NT-proBNP) levels, and renal function, the multivariate analysis found no significant association between anticoagulant type and mortality.

The retrospective study by Mentias *et al*. [16] included 551 patients with heart failure and amyloidosis who were newly diagnosed with AF between January 2015 and November 2019 and subsequently started on anticoagulation therapy. Of these, 213 received warfarin and 338 received DOACs (with apixaban accounting for 70.4% of DOAC prescriptions). Baseline characteristics were similar between the two groups. Over a median follow-up of 444 days, DOAC-treated patients had lower risks of all-cause mortality (adjusted hazard ratio [HR] = 0.71, 95% CI: 0.59–0.85), stroke (adjusted HR = 0.44, 95% CI: 0.28–0.70), and major bleeding (HR = 0.55, 95% CI: 0.36–0.84) compared to those in the warfarin group.

In an international multi-center study by Vilches *et al*. [13], data from 1191 patients with ATTR-CM were analyzed across four amyloidosis referral centers in Europe and the United States. Of these, 531 patients with AF received anticoagulation therapy with either VKAs (n = 322) or DOACs (n = 239). The incidence rates of embolic events were 2.0 per 100 patient-years (95% CI: 1.2–3.4) in the VKA group and 1.6 per 100 patient-years (95% CI: 0.7–3.9) in the DOAC group (*p* = 0.66). There was no significant difference in the risk of major bleeding between the groups (HR = 1.92, 95% CI: 0.94–3.94).

### 3.3 Quantitative Analysis: DOACs versus VKAs in Patients with CA

**Fig. 2.**
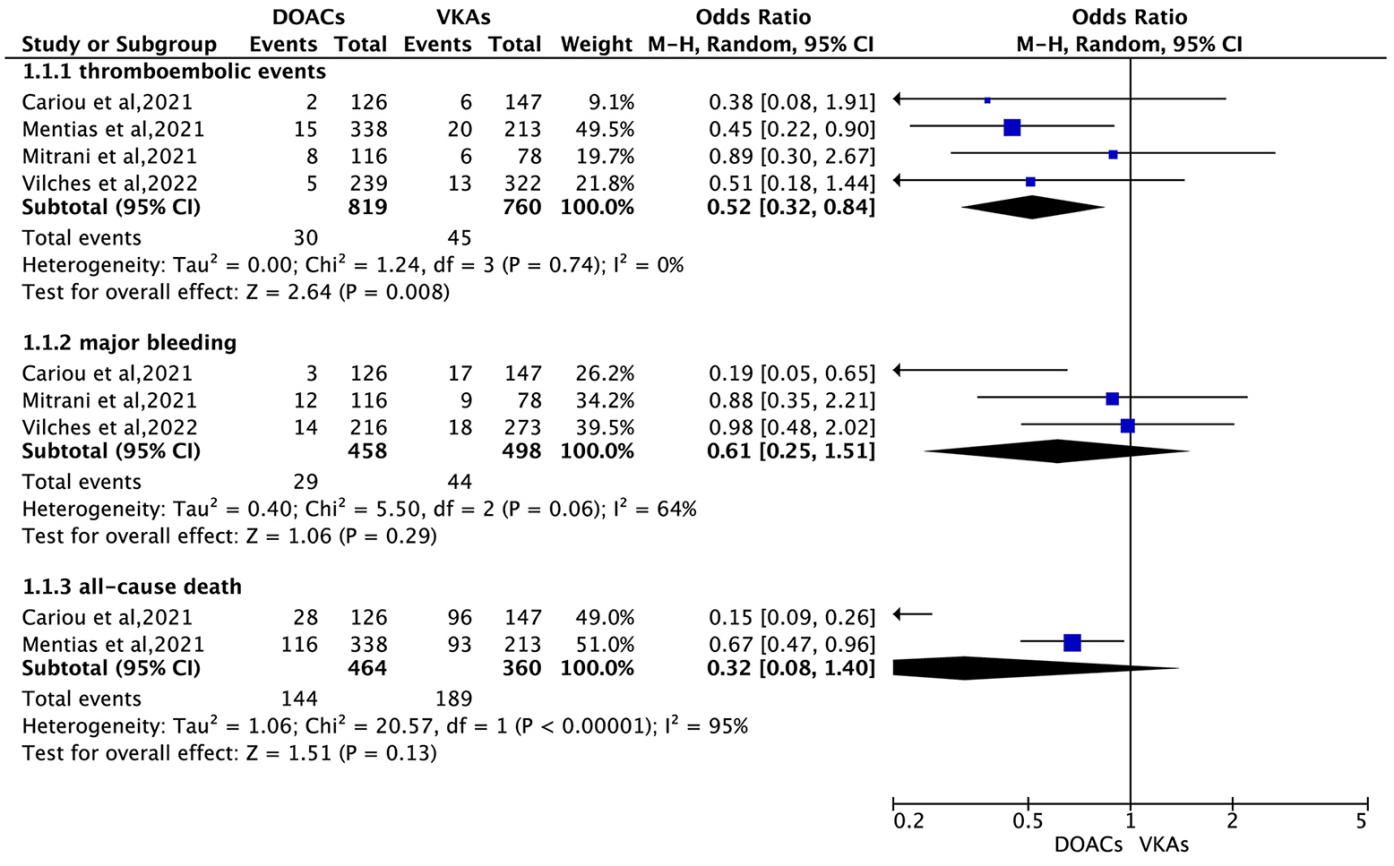
**Thromboembolic, bleeding, and all-cause death outcomes: DOACs vs. VKAs in patients with AF and cardiac amyloidosis (CA)**. Fig. 2 illustrates the pooled effects of DOACs versus VKAs in patients with AF and CA. The random-effects model was used to account for heterogeneity across studies. M-H, Mantel-Haenszel.

In the pooled analysis, results from the random-effects model demonstrated that DOACs were associated with a significantly reduced risk of thromboembolic events compared to VKAs (odds ratio [OR] = 0.52, 95% CI: 0.32–0.84). There was no significant difference between DOACs and VKAs in the risk of major bleeding (OR = 0.61, 95% CI: 0.25–1.51) or all-cause death (OR = 0.32, 95% CI: 0.08–1.40) between the two groups (Fig. 2).

## 4. Discussion

The present study investigated the effect of anticoagulation therapy—DOACs versus VKAs—in patients with CA and AF. In terms of embolic events, three of the included studies [13, 14, 15] reported no significant difference between DOACs and VKAs, while one study [16] found that DOACs were associated with a lower risk of stroke. Regarding bleeding complications, two studies [13, 14] observed similar rates between the DOAC and VKA cohorts, whereas the remaining two studies [15, 16] reported a higher frequency of bleeding events in the VKA group. In the pooled quantitative analysis, DOACs were associated with a reduced risk of thromboembolic events compared to VKAs, with no significant difference observed in the incidence of major bleeding. These findings support the safe and effective use of DOACs as a non-inferior alternative to VKAs in patients with CA and AF.

Renal function emerged as a significant confounding factor influencing outcomes in anticoagulation therapy. Patients with impaired renal function were more likely to be prescribed VKAs since DOACs such as edoxaban and dabigatran are contraindicated in patients with an estimated glomerular filtration rate (eGFR) below 15 and 30 mL/min respectively [17, 18]. Consequently, the VKA group inherently comprised individuals with poorer renal function, which predisposed them to a higher risk of bleeding. Therefore, the increased incidence of bleeding events in the VKA group may not be directly attributed to the drug itself, but rather to the confounding effects of reduced renal function. The association was underscored by the findings of Cariou *et al*. [15], where the apparent superiority of DOACs over VKAs in reducing all-cause mortality observed in univariate analysis was no longer significant after adjusting to age, cardiac, and renal function. Furthermore, in the ATTRwt subpopulation, reduced eGFR was identified as the sole variable significantly associated with all-cause mortality.

The cohort study by Mitrani *et al*. [14] included patients diagnosed prior to the Food and Drug Administration (FDA)’s approval of dabigatran, the first DOAC, in 2010. As a result, patients treated before 2010 would have received VKAs regardless of their kidney function as DOACs were not yet available. In contrast, more recent studies showed that patients with preserved renal function were more likely to be prescribed DOACs [13, 15, 16]. Therefore, the VKA arm in Mitrani *et al*.’s [14] cohort likely presented with superior renal function compared to those in newer studies, potentially contributing to the more balanced outcomes reported in this study. However, the study did not report the exact exact average eGFR for either group, limiting further comparisons.

In the study by Cariou *et al*. [15], two additional types of VKAs, fluindione and acenocoumarol, were used alongside warfarin. Fluindione is predominantly prescribed in France, accounted for 80% of VKA prescriptions in the country [19]. Fluindione is an indanedione derivative contrary to warfarin and acenocoumarol, which are coumarin derivatives [20]. It is regarded as an interesting alternative to warfarin due to its longer half-life, which might be a useful attribute in stabilizing the international normalized ratio (INR). Acenocoumarol is a drug that has some geographical tendencies, is more commonly used in specific European countries, such as Spain and the Netherlands [21], but rarely prescribed in North America or Asia. The half-life of this drug is shorter than warfarin, theoretically making adjustments of INR more challenging. A study suggests that warfarin’s longer half-life offers no significant clinical advantage [22], and acenocoumarol has been associated with more stable anticoagulation effects. Yet, the inclusion of these two less-studied VKAs raises the question of whether the findings achieved by Cariou *et al*. [15] could be extrapolated to warfarin or VKAs as a class, given their pharmacological differences.

AL and ATTR amyloidosis are two distinct conditions within the amyloidosis spectrum, each exhibiting distinct characteristics, particularly in terms of thromboembolic risk [23]. AL amyloidosis results from neoplastic plasma cells producing misfolded unstable free light chains [23] that could deposit in virtually any organ, and are not limited to the heart. Common extracardiac manifestations include renal failure, elevated liver enzymes, gastrointestinal symptoms, macroglossia, and autonomic dysfunction [24]. In AL patients, renal dysfunction is often a direct consequence of disease progression rather than heart failure. Nephrotic syndrome, associated with urinary loss of anticoagulant factors (e.g., antithrombin, protein S), immunomodulatory drug use, high free light chain levels, and elevated beta-2 microglobulin levels, collectively increase thromboembolic risk. At the same time, renal failure and increased vessel fragility caused by amyloid deposition elevate the risk of bleeding [25].

On the other hand, ATTR amyloidosis follows a distinct pathogenic pathway, primarily involving cardiac amyloid deposition [26]. Transthyretin, a tetrameric protein produced by the liver, functions as a transporter for thyroxine and retinol-binding protein. Mutations in the amyloidogenic genes destabilize the tetramer structure, leading to abnormal protein misfolding [27], aggregation, and fibril formation. In wild-type ATTR amyloidosis, the misfolded non-mutated transthyretin proteins assemble into soluble oligomers, which are prone to forming amyloid fibrils [27]. These insoluble fibrils accumulate in various organs, particularly in the elderly, resulting in wild-type amyloidosis [27]. Unlike AL amyloidosis, ATTR typically does not involve other vital organs or the use of immunomodulatory drugs, resulting in comparatively lower thrombotic and bleeding risks.

DOACs are contraindicated in patients receiving chemotherapy regimens containing dexamethasone. Both rivaroxaban and apixaban are primarily metabolized by CYP3A4 in the liver. Dexamethasone, a known Cytochrome P450 3A4 (CYP3A4) inducer, increases the enzyme’s expression, thereby accelerating the metabolism of these DOACs [28]. This reduces anticoagulant levels, potentially leading to subtherapeutic anticoagulation [28]. In the study by Cariou *et al*. [15], 69 patients with AL amyloidosis were anticoagulated, and only 15 receiving DOACs, indicating that AL patients were more likely to be on warfarin. While the study did not provide specific information about chemotherapy in this cohort, the potential drug-drug interaction between DOACs and dexamethasone likely contributed to this treatment pattern.

AL amyloidosis presents with higher prothrombotic and bleeding risks, explaining the greater proportion of AL patients managed with warfarin. However, warfarin is generally considered less effective and more challenging to manage compared to DOACs. In contrast, ATTRwt develops as an age-related condition [29], and its diagnosis typically occurs at a significantly older age AL amyloidosis. Chronic kidney disease (CKD) is often associated with an advanced age [30], results in poorer renal function in the ATTRwt population. Although AL amyloidosis directly impacts renal function due to amyloid deposition, the overall renal function in ATTRwt patients is often worse due to the higher prevalence of age-related CKD in this group.

DOACs offer several advantages over VKAs such as warfarin, including a wider therapeutic window, shorter half-life, and reduced need for frequent INR monitoring [31]. Maintaining a stable and therapeutic INR is a significant problem when administering VKAs in CA patients, which highly depends on the center and patient’s adherence. In the study by Mitrani *et al*. [14], 87.5% of the patients in the VKA group had a labile INR during follow-up, and all ischemic events or major bleeding episodes occurred in patients with unstable INR levels. Similarly, in the multicenter study by Vilches *et al*. [13], labile INR was observed in 18.7% of patients treated with VKAs and was associated with a higher rate of embolic events. However, limitations in data, such as missing information on INR stability and ejection fraction, reduce the credibility and generalizability of these findings.

Once considered incurable, ATTR-CM has seen significant advancements with the development of novel disease-modifying therapies over the past decades. In 2019, the FDA approved the first and only agent for ATTR-CM, tafamidis [32]. As additional promising therapies are anticipated to gain approval and enter the market, there is a growing need to evaluate potential drug-drug interactions between these emerging agents and commonly used anticoagulants. However, none of the existing studies have reported data on the use of chemotherapy or targeted therapy agents in their patient populations. Future trials would benefit from including such details in their population demographics to provide more comprehensive insights into these interactions.

### 4.1 Sub-population of AL Patients

Only one [15] of the studies included AL patients in their population, whereas all the other studies either only focused on ATTR patients [13, 14] or did not report the specific amyloidosis subtypes [16]. In the sub-population analysis of AL patients, there was no difference in bleeding events between the two anticoagulation treatments, and no association between types of anticoagulants and all-cause mortality was found.

### 4.2 Strengths and Limitations

By critically evaluating the current evidence, our review provides valuable insights for clinicians managing AF in patients with CA. This research holds particular significance as it can assist healthcare providers in making informed decisions regarding the selection and administration of anticoagulant therapy in this complex and high-risk patient population.

Several limitations of this review must be considered. First, all four included studies were retrospective in design, which introduces inherent limitations, including susceptibility to selection bias, confounding variables, poorly established temporal relationships, and the potential for low-quality data. Consequently, our findings should be interpreted with caution. Second, no randomized controlled trials (RCTs) have specifically compared DOACs and VKAs in AF patients with CA. High-quality RCTs are needed to provide more definitive evidence and to support robust clinical recommendations. Third, limited data availability prevented us from performing a subgroup analysis based on individual CA subtypes such as ATTR and AL. Fourth, advanced statistical techniques to account for key confounders, such as meta-regression, were not feasible due to the small number of studies included. Finally, due to the limited number of studies, we did not formally assess publication bias in this review. According to Cochrane guidelines, a funnel plot is generally considered appropriate when at least ten studies are available. The small sample size raises the possibility of publication bias, as unpublished studies with null or negative results may have been omitted from the literature, limiting the robustness and generalizability of our findings. Future research should include additional studies to enable formal publication bias assessments and enhance the reliability and robustness of the evidence on the safety and efficacy of DOACs versus VKAs in AF patients with CA.

## 5. Conclusions

Existing studies support the use of DOACs as a non-inferior therapeutic option compared to VKAs for preventing thromboembolism in patients with AF and CA. DOACs offer practical advantages, such as reduced monitoring requirements and a lower likelihood of labile anticoagulation. However, the evidence remains limited by the retrospective nature of current studies and the lack of RCTs. Further prospective trials are essential to confirm these findings and to establish robust clinical guidelines tailored to the unique challenges of anticoagulation in this high-risk population.

## Availability of Data and Materials

The datasets used and/or analyzed during the current study are available from the corresponding author on reasonable request.

## Supplementary Material

Supplementary material associated with this article can be found, in the online version, at https://doi.org/10.31083/RCM26948.
